# Novel Flu Viruses in Bats and Cattle: “Pushing the Envelope” of Influenza Infection

**DOI:** 10.3390/vetsci5030071

**Published:** 2018-08-06

**Authors:** Suresh V. Kuchipudi, Ruth H. Nissly

**Affiliations:** Animal Diagnostic Laboratory, Department of Veterinary and Biomedical Sciences, The Pennsylvania State University, University Park, PA 16802, USA; rah38@PSU.EDU

**Keywords:** influenza viruses, bats, cattle, *Orthomyxoviridae*

## Abstract

Influenza viruses are among the major infectious disease threats of animal and human health. This review examines the recent discovery of novel influenza viruses in bats and cattle, the evolving complexity of influenza virus host range including the ability to cross species barriers and geographic boundaries, and implications to animal and human health.

## 1. Introduction 

Influenza viruses are known to constantly evolve and cross species barriers. The genetic diversity of influenza viruses is ever increasing with more novel influenza subtypes being discovered periodically. The purpose of this review is to provide an up-to-date overview of ecology and evolution of influenza viruses including the novel influenza viruses in bats and cattle. In addition, we discussed the growing complexity of influenza virus–host interactions and highlighted the key research questions that need to be answered for a better understanding of the emergence of pandemic influenza viruses.

## 2. Influenza

Influenza is among the major infectious disease problems affecting animal and human health globally. Several human influenza pandemics have been recorded since 1590 AD [1], with the most significant of those being the “*Spanish flu*” of 1918, often referred to as the “mother of all pandemics” [2]. Spanish flu pandemic is believed to have affected approximately 25–30 percent of the world’s population and caused more than 50–60 million human deaths globally [3]. Influenza infections in humans occur either as epidemic (seasonal or interpandemic) influenza caused by influenza A and B viruses, or as sporadic pandemic influenza caused by influenza A viruses [4]. Study of influenza pandemics has been of great interest to epidemiologists. Influenza epidemics and pandemics have been repeatedly occurring for centuries, but to date the ability to predict a pandemic has not been achieved [5]. 

## 3. Influenza Viruses

Influenza viruses belong to the family *Orthomyxoviridae* (from the Greek *orthos*, meaning “standard, correct”, and *myxa*, meaning “mucus”). Members of *Orthomyxoviridae* are characterized by virions that are either spherical or pleomorphic measuring 80–120 nm in diameter. Genomes are comprised of single-stranded negative-sense RNA that is arranged in either eight, seven or six segments depending on the genus. Classification of the historically described A, B and C influenza viruses based on antigenic differences in nucleocapsid (NP) and matrix (M1) proteins. Influenza A viruses (IAVs) are further classified into subtypes based on the antigenic properties of the external glycoproteins namely hemagglutinin (HA) and neuraminidase (NA). IAVs and Influenza B viruses (IBVs) can cause severe upper respiratory disease in humans and are included in the seasonal flu vaccine for humans. Influenza C viruses (ICVs) are known to cause relatively mild disease in humans [6]. IAV can infect a broad range of hosts, whereas IBV and ICV have a relatively narrow host range. IBV has been isolated from seals, and both IBV and ICV are known to infect pigs [7]. 

IAVs are known to be species jumpers, frequently spilling over from their natural host and infecting new host species. Influenza viruses evolve by two different mechanisms namely antigenic *drift* and antigenic *shift*. Influenza virus genomes exhibit a rapid rate of mutation compared to DNA viruses due to the low fidelity of RNA-dependent RNA polymerase and lack of a proofreading mechanism. Antigenic *drift* is the process of mutations over time that alter antigenic sites so that a host immune system is no longer able to recognize them [8]. Mutation rate varies among influenza viral genes due to different selective pressures and evolutionary constraints. While genes coding for surface proteins are subjected to strong selection pressure by neutralizing antibodies of host immune systems, genes coding for internal proteins are not subjected to the same level of host immune selection pressure and are instead thought to undergo significant host-specific adaptive evolution [9].

Antigenic *shift* is the exchange of genome segments between two influenza virus subtypes resulting in a variant virus that is significantly different from both parent viruses [8]. Rarely, variant influenza viruses emerge due to reassortment and could rapidly adapt to new host environment and could cause pandemics [10,11]. While co-infection of a cell with two different influenza virus types could theoretically result in reassortment, the proteins of both viruses must be compatible for the reassortment to occur [12], so this is an uncommon event. The segmented nature of IAV genomes requires a complex coordination for the selective packaging of the eight-distinct viral ribonucloeprotein complexes (vRNPs) into a progeny virion during replication [13]. All eight genome segments of IAVs carry packaging signals [14] which, through RNA-RNA or RNA-protein interaction networks, mediate the formation of eight different vRNPs that are packaged into a virion [15,16,17,18].

## 4. Expanding Host Range of IAVs

IAVs are known to infect humans and a wide variety of animals including pigs, horses, minks, seals, whales and birds [19]. Avian influenza viruses (AIVs) continue to spread, evolve and cause significant economic losses to poultry industries globally. Unlike low pathogenic avian influenza viruses (LPAIVs) that cause mild clinical signs in domestic poultry, infection of gallinaceous poultry with highly pathogenic avian influenza viruses (HPAIVs) results in severe fatal disease often causing up to 100% mortality within two-three days [20]. AIVs have also been implicated in the generation of influenza viruses that have caused all human influenza pandemics. In particular, human infection with certain contemporary Eurasian lineage HPAI H5N1 viruses frequently caused severe disease with a case fatality rate of up to 50% [21]. 

Ability to infect a wide range hosts is a key contributing factor to the complex and seemingly expanding genetic diversity of IAVs. It is now well established that IAVs infect domestic pets such as dogs and cats, adding to the list of host species that could potentially expose humans to influenza viruses. An H3N8 equine influenza virus in 1999 and an avian virus-like H3N2 strain around 2005 or 2006 were transmitted to dogs, and these canine influenza viruses have been circulating in the U.S. dog population ever since [22]. After the first report of pandemic H1N1 influenza A virus (pH1N1) infection of cats in Italy in 2011 [23], more than 500 cats became infected with influenza subtype H7N2 in animal shelters in New York, NY, USA during 2016–2017 [24]. While transmission of these animal viruses to humans has not yet been documented, the close contact of many humans with pet dogs and cats presents an increased risk of opportunity for such a host jump. Transmission of IAVs between animals and humans is bidirectional such that humans are infected by animal influenza viruses and human influenza viruses have been shown to transmit to animals. In particular, studies established that domestic pets are susceptible to infection by human influenza viruses. For example, dogs experimentally inoculated with a human seasonal H3N2 or pandemic (pdm) H1N1 (2009) showed nasal shedding and seroconversion. However, dogs inoculated with influenza B virus did not exhibit virus shedding or seroconversion highlighting that interspecies transmissibility is a key feature of IAVs and not IBVs [25]. 

## 5. Novel Influenza Viruses in Bats

Aquatic birds such as ducks, gulls, and shorebirds have historically been identified as the primary reservoir of all the known IAV subtypes [26]. Recently, Northwest Atlantic gray seals were suggested as an endemically infected wild reservoir population for diverse influenza viruses [27]. Arguably, influenza virus host range could be much broader than currently known, with additional reservoirs that are yet to be revealed. Prior to 2011, 16 antigenically different HA (H1–H16) and nine different NA (N1–N9) types had been described, all of which were found in the aquatic bird reservoir. However, the number of HA and NA types have now expanded after the identification of two novel influenza-like viruses in fruit bats. The first was in a frugivorous yellow-shouldered bat (*Sturnira lilium*) in Guatemala [28]. Soon after, a second virus was found in the flat-faced fruit bat (*Artibeus planirostris*) in Peru [29]. The newly identified bat flu viruses are genetically distinct from all previously known IAVs and hence are designated as novel subtypes namely H17N10 and H18N11 [29]. Serological surveys showed wide spread prevalence of H17/H18-specific antibodies among bat populations in Central and South America [29]. 

Finding novel bat-influenza viruses is not surprising as bats represent 24% of all known mammalian species [30] and have been known to be natural reservoirs of many deadly zoonotic RNA viruses including Ebola virus [31,32,33]. It is believed that bats have the capacity to harbor more influenza virus genetic diversity than all the other mammalian and avian species combined [29]. Notably, little yellow-shouldered bats in Central America have been proposed as a potential sylvatic mammalian reservoir of influenza [28]. 

## 6. Role of Bats in the Ecology and Evolution of IAVs

Discovery of novel bat IAVs raises numerous questions including the host range evolution of IAVs and the role of bats in evolution of IAVs. 

The potential for the newly discovered bat IAVs to reassort with previously known IAV types is being explored and providing novel information about the IAV life cycle. Bat flu viruses have been reconstructed by reverse genetics using synthetic DNA which has allowed for more functional understanding of these viruses [34]. Multiple experimental studies have shown that reassortment of bat IAVs and conventional IAVs does not occur under experimental conditions [35,36,37]. This is partly explained by functional differences between bat IAVs and conventional IAVs. IAV nonstructural protein NS1 is a multifunctional protein that plays a crucial role in evading host immune responses and serves as a key virulence factor [38,39]. Unlike the NS1 of conventional IAVs, bat NS1 fails to bind to the host p85β, a regulatory subunit of the cellular metabolism-regulating enzyme phosphoinositide 3-kinase (PI3K) [40]. In addition, the surface glycoprotein HA and NA genes of bat IAVs only show low nucleotide sequence identity with those of the conventional IAVs [28,29]. Notably, it has been found that the bat IAV hemagglutinin (HA) and neuraminidase (NA) proteins lack the receptor binding activity that are characteristic of the conventional IAV [28,41]. HA of bat IAVs does not bind to the classical avian (SAα2,3-Gal) or human (SAα2,6-Gal) IAV receptors highlighting the possibility of unique entry mechanisms that are yet to be identified [42]. Consequently, it was found that bat IAVs initiate infection at the basolateral membrane of cells unlike conventional IAVs which preferential initiate infection on the apical surface of cells [34]. These findings highlight that there are major evolutionary constraints to bat IAVs to effectively reassert with conventional IAVs and/or infect other species including humans. 

## 7. Bats as “Mixing Vessels” of IAVs

While the zoonotic potential of bat IAVs is not yet fully established and continues to be a focus of ongoing research, bats have been found to be susceptible to infection by conventional IAVs. A recent study found serological evidence of IAV H9 subtype infection in 30% of frugivorous bats tested in Africa [43]. A key source of IAV genetic diversity could come from the replication of IAVs in a non-native host species that initiate evolution of new virus variants [44]. The receptors for previously known IAVs are sialic acids (SA) on host cells [45]. Consequently, the expression of these receptors on host cells is a key determinant of the ability of IAVs to infect a host [46]. AIVs preferentially bind to SA receptors that are linked to galactose by an α 2,3 linkage (SAα2,3-Gal), while human and classical swine influenza viruses show preference to α2,6 linked SAs (SAα2,6-Gal). It is widely believed that hosts that co-express both SAα2,3-Gal and SAα2,6-Gal receptors could support reassortment of IAVs and hence play a major role in the evolution of IAVs [45,47]. We have demonstrated abundant co-expression of both SAα2,3-Gal and SAα2,6-Gal receptors that are compatible with avian and human IAV binding in respiratory and digestive tracts of little brown bats, the most widespread bat species in North America [48]. The potential for bats to support infection by multiple conventional IAV types and thus serve as a source of novel genetic variants remains to be fully explored.

## 8. Novel Flu Viruses in Cattle and Reclassification of *Orthomyxoviridae*

Although influenza viruses infect humans and a wide range of animals and birds, cattle were never considered to be susceptible to influenza virus infection. However, a novel influenza virus has recently been identified in several animals including swine, cattle, sheep, and goats. The virus was first isolated as an influenza C-like virus from pigs with respiratory illness in Oklahoma, USA, in 2011 [49,50]. The virus was subsequently classified as influenza D virus (IDV), and the virus has now been reported from many countries including United States, France [51], Italy [52], China [53], Japan [54] Ireland [55] and countries in North and West Africa [56].

The International Committee on Taxonomy of Viruses (ICTV), which is responsible for developing, refining, and maintaining a universal virus taxonomy, has recently released revised classification of *Orthomyxoviridae* [57]. In earlier classification, Orthomyxoviruses belonged to any one of the five genera: *Influenzavirus A*, *Influenzavirus B, Influenzavirus C*, *Thogotovirus* and *Isavirus* [58]. However, the most recent classification of *Orthomyxoviridae* includes eight genera and nine species (Table 1). Genus *Alphainfluenzavirus* comprises the species *influenza A virus*, *Betainfluenzavirus* comprises the species *influenza B virus*, *Gammainfluenzavirus* comprises the species *influenza C virus*, and *Delatinfluenzavirus* comprises the species *IDV* [58].

## 9. Properties of IDVs

Like other influenza viruses, IDVs possess a negative-sense single-stranded RNA genome. However unlike IAVs that contain 8 segments, IDVs comprise seven genomic segments that are predicted to encode nine proteins, including glycoprotein hemagglutinin-esterase fusion (HE), polymerases PB2, PB1, and P3, nucleoprotein, matrix protein (M1 and CM2), and nonstructural proteins (NS1 and NEP) [59]. IDVs use 9-*O*-acetylated sialic acid as their cellular receptor on host cells such as ICVs [60]. 

## 10. Animal and Human Health Significance of IDVs

It is well established based on several epidemiological studies that cattle are the primary reservoirs of IDVs [50,59]. In addition, IDVs have also been isolated from a range of animals including pigs, sheep, goat, horses and camelids. While the precise role of IDVs in clinical disease in animals is not yet fully investigated, their role in causing respiratory infections in cattle has been implied. Two recent studies carried out metagenomic characterizations of the virome associated with bovine respiratory disease in feedlot cattle and found correlation of IDV presence with Bovine respiratory disease (BRD) clinical signs, raising exciting new prospects for understanding and combatting this complicated disease [61,62]. 

BRD complex is one of the major diseases affecting the cattle industry in the USA and around the world. Productivity losses due to BRD are estimated to be $23.60 per calf with an annual economic impact of more than one billion dollars to the U.S. cattle industry [61]. BRD is associated by multiple pathogens and accounts for approximately 70–80% of the morbidity in the USA [63] and 84.5–99.9% of the morbidity in Mexican feedlot cattle. BRD results in the use of widespread therapeutics and antibiotics in feedlots, which increasingly raises public health concerns of promoting antibiotic resistance [64,65]. Pathophysiology of BRD involves complex interactions between host, pathogen, environment and management factors. In feedlot cattle, BRD is initiated by viral infection followed by stress due to travel which is typically followed by a secondary infection by resident bacteria [66]. Viral infection can cause increased susceptibility to secondary bacterial infections by either immunosuppression or by damaging the epithelium of upper airways and injuring lung parenchyma which facilitates the migration of bacterial pathogens and colonization of the lower respiratory tract. Depending on several factors, the clinical outcome of BRD can be variable; however, higher morbidity and mortality are observed in the event of mixed viral and bacterial infections [67].

Many viral pathogens have been implicated in BRD, which include bovine viral diarrhea virus, bovine herpesvirus 1, bovine respiratory syncytial virus and bovine parainfluenza 3. Experimental studies in calves with IDV showed damage that results in the induction of inflammation in the trachea. IDV could be a significant player in BRD and could facilitate coinfections with other bovine pathogens [59]. In addition to the animal health implications of IDVs, a recent study found IDV antibodies in 34 out of 35 persons that had contact with cattle and only 2 out of 11 that did not have any exposure to cattle [68]. The results of this study raised the possibility that IDV could be relevant from a public health stand point and that it could pose a zoonotic risk to cattle-exposed workers. With much still to be characterized about the new IDV species, exploring its impact on human an animal health through epidemiological studies will be vital to understanding spread of this virus.

## 11. Conclusions

Emerging and novel zoonotic infections often result from pathogens jumping from their original host into novel host species [69]. The host range evolution of mammalian viruses typically involves more closely related hosts [70,71]. In particular, RNA viruses with broad host range are more likely to jump between distantly related species [70,72,73]. A key determinant in the host range evolution of a virus is the mechanism of viral entry used by the viruses. Analysis of 64 human viruses revealed that the viruses that use receptors that are highly conserved in their amino acid sequence across species have the broadest host range [74]. The ability of viruses to bind to an alternative receptor is sometimes key in species jumping. AIVs need to change to preferentially bind to SAα-2,6-Gal receptor to efficiently transmit between humans. In experimental studies, it was shown that the shift from SAα-2,3-Gal to SAα-2,6-Gal binding requires four mutations for a HA of HPAI H5N1 viruses [75]. However, several newly emerged H5N1 AIVs in Egypt have been found to have acquired the human receptor SAα-2,6-Gal binding ability during their emergence in birds [76].

**Figure 1:**
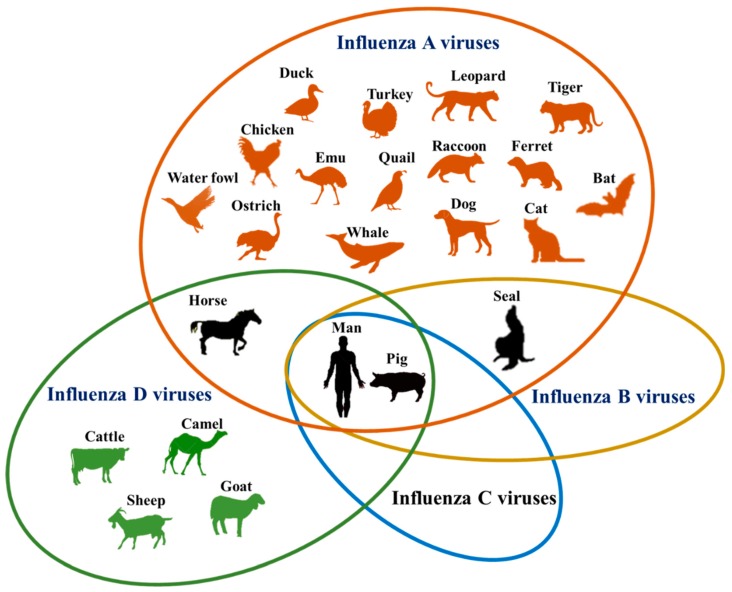
Host range of influenza viruses by species. Common hosts of more than one species are encompassed in overlapping ovals. Of the numerous hosts which support influenza virus infection, only four (horse, seal, man and pig) are known to be susceptible to more than one species.

Influenza A, B, C and D viruses have varying susceptible host range (Figure 1). Notably, the newly discovered IDVs have the widest host range after IAVs. Further, humans and pigs are susceptible to infection by all four types of influenza viruses. With the extensive host range that continues to grow and the zoonotic potential, influenza viruses remain a major challenge to epidemiologists. Owing to their tremendous potential to affect animal and human health, there is a need to carry out in-depth and comprehensive studies to unravel the ecological complexity of influenza virus host range evolution. In particular, we feel that researchers should focus on answering the key questions, “what is the role of bats in the ecology and evolution of IAVs?”, “are IDVs involved in the epidemic influenza infections in people?”, and “are birds susceptible to infection by IDVs?” 

## Figures and Tables

**Table 1:** Revised classification of *Orthomyxoviridae* (ICTV 2017).

Genus	Species	Genomic Segments
Alphainfluenzavirus	Influenza A virus	8
Betainfluenzavirus	Influenza B virus	8
Deltainfluenzavirus	Influenza D virus	7
Gammainfluenzavirus	Influenza C virus	7
*Isavirus*	Salmon isavirus	8
Quaranjavirus	Johnston Atoll quaranjavirus	6
Quaranfil quaranjavirus		
Thogotovirus	Dhori thogotovirus	6
Thogoto thogotovirus		
